# Systematic review and network meta-analysis to compare vaccine effectiveness against porcine edema disease caused by Shiga toxin‐producing *Escherichia coli*

**DOI:** 10.1038/s41598-022-10439-x

**Published:** 2022-04-19

**Authors:** Sim-In Lee, Eurade Ntakiyisumba, Gayeon Won

**Affiliations:** College of Veterinary Medicine, Jeonbuk National University, 79 Gobong-ro, Iksan-si, Jeollabuk-do, 56443 Republic of Korea

**Keywords:** Microbiology, Zoology, Vaccines

## Abstract

The comprehensive effect size of several commercial vaccines and vaccine candidates against edema disease (ED) has not been evaluated to date. To integrate the effectiveness of ED vaccines reported so far and to compare and evaluate the posterior-effect estimates of each vaccine type with network models, we identified eligible studies (n = 12) from the electronic databases using specified search strings. Data for dichotomous outcomes (i.e., mortality and clinical symptoms) and continuous outcomes (i.e., fecal shedding and average daily gain) were extracted and analyzed. Conventional meta-analysis shows that, compared with that in non-vaccinated pigs, vaccinated animals are likely to show reduced mortality (OR = 0.07) and clinical signs of ED (OR = 0.11), and increased productivity (SMD = 0.73). Although reduced fecal shedding (SMD = − 1.29) was observed in vaccinated pigs, this could not be fully determined on insufficient grounds. In contrast to mortality and clinical symptoms, fecal shedding (*I*^2^ = 88%) and average daily gain (*I*^2^ = 85%) showed immense heterogeneity, which was attributed to the small sample size and vaccination route, respectively. According to the Bayesian network meta-analysis, the plasmid-based DNA vaccine demonstrated a better effect for all outcomes compared to other types of vaccines. However, these findings should be carefully interpreted with consideration to potential mediators, insufficient data, and inconsistent network models.

## Introduction

Edema disease (ED) is a bacterial disease of swine caused by Shiga toxin-producing *Escherichia coli* (STEC) that secrete Shiga toxin 2e (Stx2e)^1^. As ED affects post-weaned rapidly growing pigs, it results in tremendous economic losses to the swine industry^2^. The major virulence factors of STEC are F18ab fimbriae and Stx2e^1^. F18ab allows STEC to adhere to the surface of enterocytes, resulting in bacterial colonization in the small intestine, whereas Stx2e is an exotoxin that causes systemic vascular damage, resulting in soft tissue edema, serous effusions, or septicemia^1,3^. Consequently, gross ED lesions include edema in the eyelids, throat, subcutaneous tissue, and intestinal tract, and microscopic lesions comprise arteriolar necrosis in the brain and intestinal tract^3,4^. Systemic vascular damage in the brainstem with edema, infarction, and malacia causes neurological disorders, leading to high mortality^3,5^.

Vaccination against F18ab fimbriae or Stx2e is used to prevent ED, as it can reduce the use of antibiotics and improve animal welfare by preventing STEC infection^3,6^. The F18ab fimbriae-targeted vaccine prevents bacterial colonization in the intestinal tracts and consequently reduces the quantity of bacterial toxin entering systemic circulation^7^. The Stx2e-targeted vaccine aims to alleviate the severity of ED by generating neutralizing anti-Stx2e antibodies^3^. Furthermore, vaccination strategies that vary according to the route, dose, or frequency affect the magnitude of the immune response^8^. A systematic review and meta-analysis can help to measure and evaluate the effect size of vaccination effectiveness against STEC infection in pigs by considering the variability among previously published relevant studies.

Meta-analysis is a methodology that can investigate the variability between experiments and evaluate the generalizability of the conclusions. It can be used to increase the sample size and statistical power and improve the estimate of the effect size of vaccine effectiveness^9^. Among the various methodologies of meta-analysis, subgroup analysis can help to categorize studies based on a particular trait and compare the effects between subgroups. Further, network meta-analysis allows us to compare multiple treatments simultaneously by using direct and indirect evidence obtained from multiple interventions and by incorporating the results of multiple pairwise comparisons^10^. Scientific evidence obtained through meta-analysis can be summarized through systematic reviews using defined and transparent regulations^11^. Therefore, systematic reviews and meta-analyses of previous studies can provide deeper insights by gathering and summarizing evidence of the comparative effectiveness of vaccinations.

The main purpose of this paper was to systematically review the published studies on the effectiveness of vaccines in preventing ED and to generate a quantitative estimate of the effect size using a meta-analysis. Furthermore, this paper aimed to compare the effectiveness of vaccines against ED to provide clear guidance on decision making by farmers, clinicians, and vaccine-manufacturing companies.

## Results

### Study selection

**Figure 1:**
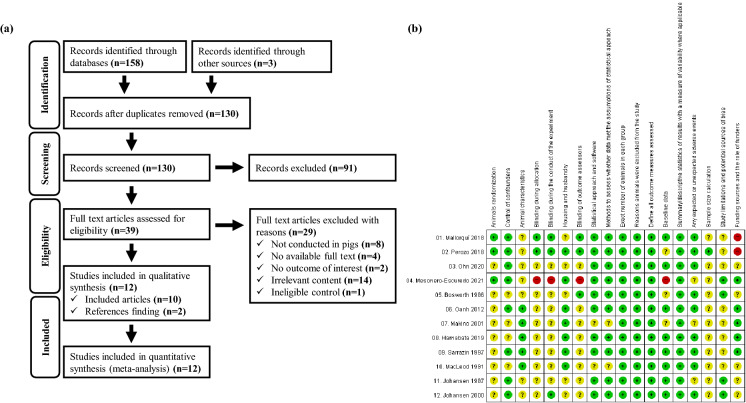
(**a**) PRISMA-P flowchart and (**b**) risk of bias assessment of eligible studies on vaccination against edema disease in pigs.

The database and gray literature searches identified 161 documents. After removing duplicates, 130 electronic records remained. A further 91 publications were excluded after the title/abstract eligibility screening, and 10 publications suitable for data extraction remained after full-text screening. Two records were identified by citation searches and reference list screening, resulting in a total of 12 publications (Fig. 1a).

### Study characteristics and outcomes

The characteristics of the 12 studies containing 31 trials are summarized in Supplemental Table S1^12–23^. In brief, four studies that evaluated the efficacy of commercial vaccines^12–15^ and eight studies that evaluated the efficacy of experimental vaccine candidates were included^16–23^. Of these, seven studies included the experimental challenge model^12,16–20,23^, and five were designed using the observation field model^13–15,21,22^. Both commercial and experimental vaccines have established Stx2e as the primary target. All commercial vaccines were recombinant toxoid vaccines that were administered intramuscularly^12–15^, whereas experimental vaccine candidates had been developed using various vaccine types (i.e., recombinant toxoid vaccine^16,17^; plasmid vaccine^18,19^; live attenuated vaccine^23^; toxoid vaccines^20,21^; anti-serum^20,22^) and administration routes (i.e., subcutaneous^16^, intramuscular^17,21,22^, oral^18,19,23^, and intraperitoneal^20^).

Fecal shedding of STEC was reported in only two studies^19,23^, whereas three outcomes, mortality^12–18,20–22^, clinical symptoms^12–17,19,20^, and average daily gain (ADG)^12–18,21^, were reported in most studies. In addition, the intake information of crude protein and antimicrobials as confounding factors were reported in only four^16,18–20^ and five studies^14,15,19,22,23^, respectively. Thus, this information was not used for subgroup analysis.

### Risk of bias within studies

The results of the risk of bias assessment are shown in Fig. 1b. Three studies^12,13,15^ were judged to be at "low" risk of bias for animal randomization. Other studies that did not show a specific randomization strategy were judged to be at "unclear" risk of bias^14,16–23^. As the susceptibility to ED is considered a main confounding factor, experimental studies on piglets not infected with ED were judged to have a “low” risk of confounding bias^12,17,23^. For the field trials, piglets with a history of ED were judged to have a “low” risk of confounding bias as exposure to STEC continuously occurred in the target animal in the field study^13–15,21,22^. Five studies that reported animal status were judged to be at "low" risk of bias for animal characteristics^17–20,23^.

Studies that clearly stated how to perform blind allocation^12,13^, blind experiment conduction^12,13,22^, and blind assessment^12,13,16^, were judged to be at "low" risk of bias for blinding items. A study in which the pigs were not allocated randomly between groups and the farm staff conducted experiments and evaluations during the entire experiment was judged to be “high” in the risk of bias due to blinding of experiment^15^. Seven studies that reported well-controlled environmental factors to avoid affecting outcomes were classified as "low" risk of bias for housing and husbandry^13,15,17–20,23^, whereas other studies that did not report environmental factors were classified as "unclear" risk of bias^12,14,16,21,22^. Except for the two studies judged to be at “unclear risk” of bias due to the lack of clear statements on statistical approaches used to analyze their data^18,20^, others that clearly described statistical approach and software were classified as "low" risk of bias^12–17,19,21–23^.

All studies presented exact sample sizes, and pigs were only excluded from the groups when there were clear reasons^12–23^. Eight studies, providing health status or age, were classified as “low” risk bias for baseline data^12,14,17,19–23^. On the other hand, one study conducted on a farm where respiratory diseases were present was classified as “high” risk of bias owing to baseline data^15^. Data from all experiments were reported with descriptive statistics in all studies^12–23^. Nine studies that evaluated the adverse effects of vaccines in all subjects were classified as “low” risk of bias for selective reporting of adverse events from vaccination^12–14,16,17,19,20,22,23^. One study that measured the minimum sample size was classified as "low" risk of bias for sample size calculation and the others were classified as "unclear" risk of bias for sample size calculation^13^. Four studies were judged to be at “low” risk of bias for limitations and potential sources as specific limitations affecting the outcome do not exist^15,16,21,22^. Studies that declared no competing interest of the authors were judged to be at “low” risk of bias for the role of the funder^15,17–19,23^, whereas two studies that were conducted by vaccine manufacturers, were judged to be at “high” risk of bias for funding sources^12,13^.

### Results of the conventional meta-analysis

#### Mortality

**Figure 2:**
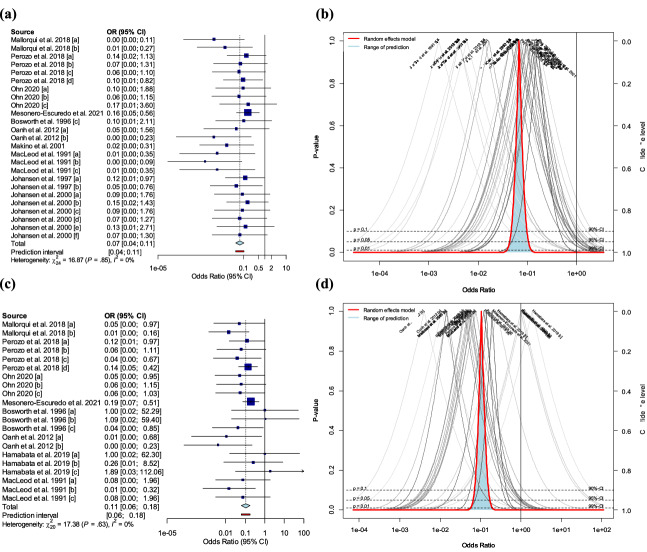
Results of conventional meta-analysis of edema disease-related mortality and clinical symptoms in pigs. (**a**) Forest plot of the odds ratio (OR) and 95% confidence interval for mortality from edema disease in pigs. Size of the blue square corresponds to the weight of each study. Vertical dotted and solid lines symbolize the overall effect size and the null value, respectively. A random-effects model was used. (**b**) Drapery plot showing *p*-value curves for mortality of edema disease. A random-effects model was used. (**c**) Forest plot of the OR and 95% confidence interval for clinical symptoms of edema disease in pigs. Size of the blue square corresponds to the weight of each study. Vertical dotted and solid lines symbolize the overall effect size and the null value, respectively. A random-effects model was used. (**d**) Drapery plot showing *p*-value curves for clinical symptoms of edema disease. A random-effects model was used. The statistical computer program R (version 4.1.1) was used to prepare this figure^44^.

Ten studies, which comprised 25 trials, evaluated the effectiveness of STEC vaccines based on mortality. The pooled odds ratio (OR) was 0.07 with 95% confidence intervals (CIs) of 0.04 to 0.11 (Fig. 2a). The pooled OR of mortality suggests that vaccinated pigs were significantly less likely to die from ED compared to non-vaccinated pigs (*p* < 0.0001). Studies evaluating pig mortality showed less heterogeneity between^2^ studies (τ^2^ = 0.00 [0.00; 0.74]; *p*-value of Q-test = 0.85; *I*^2^ value = 0% [0.0%; 0.4%]). The prediction intervals (PIs) ranged from 0.04 to 0.11, indicating that the new subsequent observation would be within this positive preventive effect less than the null value of 1. In addition to the 95% CIs, the odds of mortality in vaccinated pigs were significantly lower than that in non-vaccinated pigs at 99% CIs (0.07 [0.04; 0.13] (*p* < 0.0001)) (Fig. 2b). Subgroup analyses of mortality were not performed because of low heterogeneity. Among the existing studies evaluating mortality, nineteen trials were located in regions significantly lower than 1 in the funnel plot (*p* < 0.1), whereas six trials lie in the non-significant region (*p* > 0.1) (Fig. 4a). As a result, the missing studies of mortality were located in the region that was not significant with null value (Fig. 4a). This asymmetrical distribution in the funnel plot suggested a potential risk of publication bias in mortality data (*p*-value of Egger’s test < 0.0001). The adjusted OR after trim-and-fill method was 0.11 [0.06; 0.20] (*p* < 0.0001), which implies that the pooled OR value of 0.07 (*p* < 0.0001) in mortality was measured lower than true effect size due to the small study effect.

#### Clinical symptoms

Eight studies, which incorporated 21 trials, assessed the effectiveness of STEC vaccines based on clinical symptoms. The pooled OR was 0.11 with 95% CIs of 0.06 to 0.18, suggesting a significant (*p* < 0.0001) reduction in clinical symptoms in vaccinated pigs compared with that in non-vaccinated ones (Fig. 2c). The studies evaluating the clinical symptoms showed less heterogeneity between studies (τ^2^ = 0.00 [0.00; 1.91]; *p*-value of Q-test = 0.63; *I*^2^ value = 0% [0.0%; 0.5%]). The PIs ranged from 0.06 to 0.18, and indicated that the new subsequent observations would be within this positive preventive effect with less than a null value of 1. In addition to the 95% CIs, the odds of the occurrence of clinical symptoms in vaccinated pigs were significantly lower than those in non-vaccinated ones at 99% CIs (0.11 [0.05; 0.21] (*p* < 0.0001)) (Fig. 2d). Subgroup analyses of mortality were not performed because of low heterogeneity. The studies evaluating clinical symptoms were symmetrically distributed between significant and non-significant regions in the funnel plot, with a *p*-value of Egger’s test of 0.24 (Fig. 4b).

#### Fecal shedding

**Figure 3:**
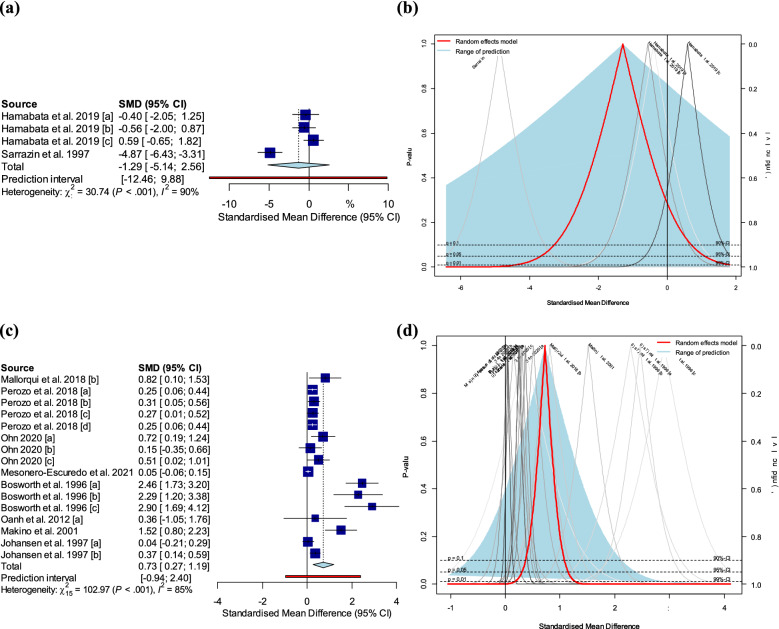
Results of conventional meta-analysis of edema disease-related fecal shedding and average daily gain (ADG) in pigs. (**a**) Forest plot of the Hedges’ g and 95% confidence interval for fecal shedding of Shiga toxin-producing *Escherichia coli* (STEC) in pigs. Size of the blue square corresponds to the weight of each study. Vertical dotted and solid lines symbolize the overall effect size and the null value, respectively. A random-effects model was used. (**b**) Drapery plot showing *p*-value curves for fecal shedding of STEC. A random-effects model was used. (**c**) Forest plot of the Hedges’ g and 95% confidence interval for ADG in pigs. Size of the blue square corresponds to the weight of each study. Vertical dotted and solid lines symbolize the overall effect size and the null value, respectively. A random-effects model was used. (**d**) Drapery plot showing *p*-value curves for ADG. A random-effects model was used. The statistical program R (version 4.1.1) was used to prepare this figure^44^.

Two studies, comprised of four trials, assessed the effectiveness of STEC vaccines based on STEC fecal shedding. The pooled Hedges’ g statistic of fecal shedding was − 1.29 with 95% CIs of − 5.14 to 2.56 (Fig. 3a). This suggests that fecal shedding was not significantly reduced in vaccinated pigs compared with that in non-vaccinated ones. The two studies were significantly heterogeneous (τ^2^ = 5.28 [1.30; 81.15]; *p*-value of Q-test < 0.0001; *I*^2^ value = 90.2% [78.0%; 95.7%]). The PIs ranged from *g* = − 12.46 to 9.88, indicating that negative intervention effects could not be excluded from subsequent observations. In addition, the fecal shedding in vaccinated pigs was not significantly higher than that in non-vaccinated pigs at the 90% CIs (Fig. 3b). Subgroup analyses and assessment of the publication bias were not performed because of the low number of studies (n = 4).

#### Average daily gain

**Figure 4:**
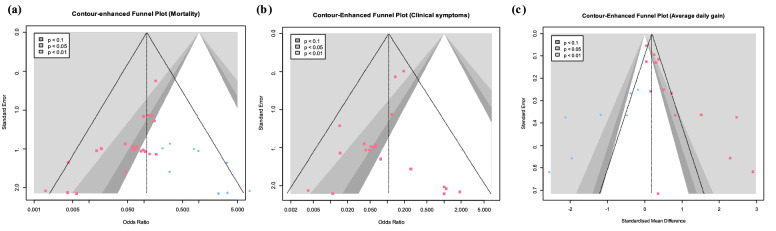
Contour-enhanced funnel plots of eligible studies for each outcome: (**a**) mortality, (**b**) clinical symptoms, and (**c**) average daily gain. Dots represent the individual study, whereas filled red rectangles and filled blue circles represent existing studies and missing studies adjusted by trim-and-fill method, respectively. The statistical program R (version 4.1.1) was used to prepare this figure^44^.

Eight studies, comprised of 16 trials, evaluated the effectiveness of STEC vaccines based on ADG. The pooled Hedges’ g of ADG of 0.73 with 95% CIs of 0.27 to 1.19 suggested a significant (*p* < 0.01) increase in ADG for vaccinated pigs compared with that in non-vaccinated ones (Fig. 3c). These studies were substantially heterogeneous (τ^2^ = 0.56 [0.28; 1.87]; *p*-value of Q-test < 0.0001; *I*^2^ value = 85.4% [77.8%; 90.4%]). The PIs ranged from *g* = − 0.94 to 2.40, indicating that negative intervention effects could not be excluded from subsequent observations. In addition to the 95% CIs, the Hedges’ g of ADG in vaccinated pigs was significantly elevated than the null value of 0 at 99% CIs (0.73 [0.09; 1.36] (*p* < 0.01)) (Fig. 3d). The results reported by Bosworth et al.^16^ and Mesoreno-Escuredo et al.^15^ were outside the 95% CIs of the pooled Hedges’ g. The results of the leave-one-out method showed that the findings of Bosworth et al. substantially influenced the pooled effect size and heterogeneity; therefore, this study was judged to be an influential outlier (Supplemental Fig. S1). The results of the meta-analysis of ADG without the influential outlier are summarized in Supplemental Table S2. After the removal of the influential outlier, the heterogeneity was reduced to 65%. The results of the subgroup analysis are summarized in Supplemental Table S3. The effect sizes of vaccines between subgroups, divided by commercial availability of a vaccine (*p* < 0.05), study types (*p* < 0.01), vaccine types (*p* < 0.01), and route of vaccination (*p* < 0.0001), showed a statistically significant association. In contrast, there were no significant differences between subgroups based on the growth stage of pigs (*p* = 0.05). Subgroup analysis based on ingestion of antimicrobial or crude protein could not be performed because more than 10 of the 15 trials did not report related data. Among the existing studies evaluating ADG, 12 trials were located in regions significantly higher than the null value of 0 in the funnel plot (*p* < 0.1), whereas 4 trials were located in the non-significant region higher than 0 (*p* > 0.1) (Fig. 4c). Thus, the missing studies for ADG were evenly distributed between the significant and non-significant regions less than the null value of 0 (Fig. 4c). This asymmetrical distribution in the funnel plot suggested a potential risk of publication bias in ADG data (*p*-value of Egger’s test < 0.0001). The adjusted Hedges’ g after trim-and-fill method was 0.19 [− 0.37; 0.75] (*p* = 0.49), which implied that the pooled Hedges’ g value of 0.73 (*p* < 0.01) for ADG was measured higher than true effect size due to the small study effect.

### Results of network meta-analysis

**Figure 5:**
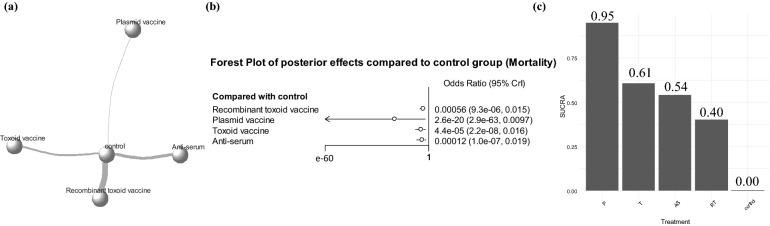
Results of network meta-analysis of edema disease-related mortality in pigs. (**a**) Network plot for mortality. Each circle represents a vaccine type, and width of lines between circles indicates number of pairs. (**b**) Forest plot of posterior effects compared to control group based on their odds ratio (OR). (**c**) Surface under the cumulative ranking curve (SUCRA) plot for mortality. P, plasmid-based DNA vaccine; T, toxoid vaccine; AS, anti-serum; RT, recombinant toxoid vaccine. The statistical program R (version 4.1.1) was used to prepare this figure^44^.

**Figure 6:**
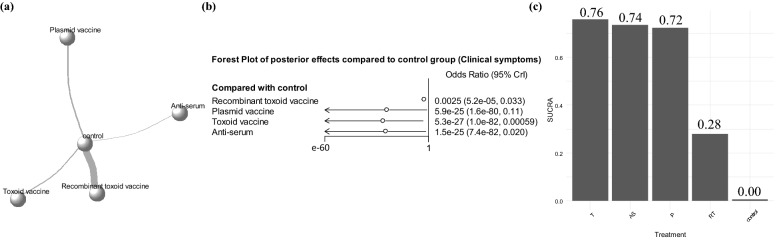
Results of network meta-analysis of edema disease-related clinical symptoms in pigs. (**a**) Network plot for clinical symptoms. Each circle represents a vaccine type, and width of lines between circles indicates number of pairs. (**b**) Forest plot of posterior effects compared to control group based on their odds ratio (OR). (**c**) Surface under the cumulative ranking curve (SUCRA) plot for clinical symptoms. T, toxoid vaccine; AS, anti-serum; P, plasmid-based DNA vaccine; RT, recombinant toxoid vaccine. The statistical program R (version 4.1.1) was used to prepare this figure^44^.

**Figure 7:**
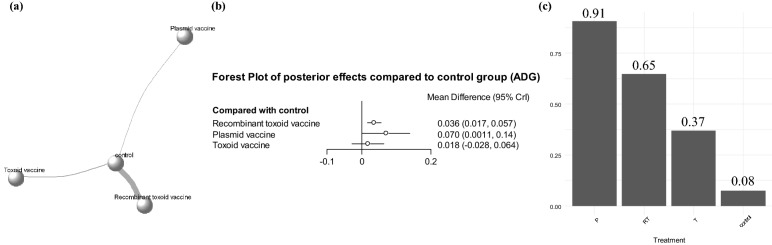
Results of network meta-analysis of average daily weight gain (ADG) in pigs. (**a**) Network plot for ADG. Each circle represents a vaccine type, and width of lines between circles indicates number of pairs. (**b**) Forest plot of posterior effects compared to control group based on their mean difference (MD). (**c**) Surface under the cumulative ranking curve (SUCRA) plot for ADG. P, plasmid-based DNA vaccine; RT, recombinant toxoid vaccine; T, toxoid vaccine. The statistical program R (version 4.1.1) was used to prepare this figure^44^.

As there were no studies that compared the effectiveness of vaccines directly, star-shaped network models centered on the control group were created for data on mortality (Fig. 5a), clinical symptoms (Fig. 6a), and ADG (Fig. 7a). No direct estimates between the vaccines existed, and the inconsistency of the models was not evaluated. Network meta-analyses for fecal shedding were not performed because of the small number of studies (n = 4). The line connecting the control group and recombinant toxoid vaccine was the thickest in the network models of all outcomes because most trials compared the effectiveness of control and recombinant toxoid vaccine, whereas few trials compared the effectiveness of control and other types of vaccines (Figs. 5a, 6a, and 7a).

The posterior OR estimates of mortality obtained through Markov chain Monte Carlo (MCMC) simulation using the results of 25 trials are presented in Fig. 5b. All other vaccine classes significantly reduced the mortality from ED compared to that after no vaccination (Fig. 5b), and the surface under the cumulative ranking curve (SUCRA) score for vaccines was higher than that for no vaccines (Fig. 5c). Among the vaccine types, the plasmid-based DNA vaccine was observed to be the most effective (SUCRA score 95%; posterior OR estimate = 2.6e−20 [2.9e−63; 0.097]). The posterior OR estimates of clinical symptoms obtained through MCMC simulation using the results of 21 trials are presented in Fig. 6b. The odds of the appearance of clinical symptoms were lower in pigs immunized with any type of vaccine than in non-vaccinated pigs. In particular, the toxoid (SUCRA score 76%; posterior OR estimate = 5.3e−27 [1.0e−82; 0.00059]), anti-serum (SUCRA score 74%; posterior OR estimate = 1.5e−25 [7.4e−82; 0.020]), and plasmid-based DNA (SUCRA score 72%; posterior OR estimate = 5.9e−25 [1.6e−80; 0.11]) vaccines showed excessively low posterior OR estimates than those in the control group (Fig. 6b), and shared similar SUCRA scores (Fig. 6c).

The posterior mean difference (MD) estimates of ADG obtained through MCMC simulation using the results of 16 trials are presented in Fig. 7b. The MD with the recombinant toxoid (posterior MD estimate = 0.036 [0.017; 0.057]) and the plasmid-based DNA (posterior MD estimate = 0.070 [0.0011; 0.14]) vaccines was significantly greater than that in the control group (Fig. 7b). The MD with the toxoid vaccine (posterior MD estimate = 0.018 [− 0.028; 0.064]) was higher than that in the control group, but the 95% credible intervals (CrIs) of MD with the toxoid vaccine contained a negative range (Fig. 7b). The SUCRA score was highest for the plasmid-based DNA vaccine (91%), followed by the recombinant toxoid vaccine (65%), the toxoid vaccine (37%), and the control group (Fig. 7c). Overall, more effective posterior estimates and SUCRA scores were observed for all outcomes in pigs vaccinated with the plasmid-based DNA vaccine compared with those for the other types of vaccines.

## Discussion

In this study, the pooled effectiveness of vaccines against swine ED was investigated based on currently available data from published eligible studies (n = 12). The combined effectiveness of the vaccines against ED was assessed based on mortality, clinical symptoms, STEC fecal shedding, and ADG outcomes in vaccinated and non-vaccinated pigs. In the conventional meta-analysis, substantially higher effect sizes were observed for all outcomes in vaccinated pigs except fecal shedding than those in non-vaccinated pigs (Figs. 2b,d, and 3d). As to the ADG outcomes, the Hedges’ g of ADG in vaccinated pigs was significantly increased than the null value of 0 with 99% CIs although the 95% PIs included negative values (Fig. 3c). The results indicated that ADG outcomes may be positively affected by the vaccination against ED. With regard to fecal shedding, vaccination did not statistically influence the decrease in STEC shedding (Fig. 3a). The current evidence of fecal shedding did not fully support the hypothesis that vaccination reduces the fecal shedding rate in affected animals as both wide 95% CIs and PIs in our analysis included the null value of 0. The PIs are commonly used to predict the dispersion of the effect sizes in each individual of the population, and estimate what true effect size can be expected in the similar study in different settings^24^. The CIs are generally applied to the estimation of population parameter, representing the accuracy of the mean of effect size in the present study^24^. In summary, vaccination against ED is expected to reduce mortality and clinical symptoms of ED and to provide a beneficial effect for the increase in ADG in pigs. However, as the decrease in STEC fecal shedding in vaccinated pigs could not be confirmed due to the lack of supporting data, future meta-analysis should be conducted by collecting more data on fecal shedding.

No significant heterogeneity between studies was perceived among the observed mortality and clinical symptoms in each study (Figs. 2a,c). Thus, it is possible to accurately interpret the experimental results and predict future observations of mortality and clinical symptoms. Significant heterogeneity was observed between studies that evaluated fecal shedding and ADG (Figs. 3a,c). Due to the small number of studies (n = 4) that evaluated STEC fecal shedding, subgroup analysis could not be performed to investigate the possible sources of heterogeneity, and the risk of publication bias could not be assessed^25,26^. These limitations may hamper the formulation of a comprehensive conclusion on the effectiveness of vaccines against STEC fecal shedding. In the subgroup analysis of ADG, the explanatory power for the high heterogeneity of ADG data was highest when the group was classified by vaccination route (Supplemental Table S3). The subcutaneous and oral vaccination subgroups only included data from Bosworth et al.^16^ and Makino et al.^18^, respectively, whereas the remaining studies were included in the intramuscular vaccination subgroup^12–15,17,21^. As these two studies^16,18^ were evaluated as influential cases of heterogeneity in the influence analysis (Supplemental Fig. S1), the heterogeneity of the remaining intramuscular vaccination subgroups decreased (Supplemental Table S3). In addition to routes of vaccination, there was a statistically significant difference in effect size between subgroups categorized as study type (i.e., experimental challenge study and field trial). Both study types are part of the experimental epidemiologic research in which outcomes are measured under a controlled environment^27^. However, the study type was employed as one of the covariates necessary for conducting the subgroup analysis in this analysis as they have differences in an outline of the practical approaches^27^. In the analysis, studies conducted under the experimental challenge model showed higher vaccine efficacy on ADG compared to those in the field trials (Supplemental Table S3). The results may be affected by exposure to natural infection used in the field trial, which is not standardized. As the intakes of crude protein, crude fiber, or antibiotic among the preset subgroup categories could be also potential mediators of outcomes for vaccine evaluation, it although the heterogeneity of ADG data could be explained to some extent by the route of vaccination, a comprehensive conclusion about intermediate heterogeneity within intramuscular subgroup cannot be drawn due to the insufficient number of studies within the potential mediators subgroup^28^. was expected that the heterogeneity within the intramuscular subgroup would be explained by these potential mediators^15,29^. However, only a few studies reported the feed or antibiotic intake in pigs, and therefore this category could not be used for subgroup analysis. In summary, In future meta-analyses, heterogeneity should be analyzed by further investigating the potential mediators in multiple publications^11^.

Based on Egger’s test results and the trim-and-fill method, a potential risk of publication bias was found in the mortality outcome and ADG outcome, whereas the outcome for clinical symptoms did not show significant publication bias (Fig. 4a–c). After the results were analyzed by the trim-and-fill method, the adjusted mortality OR did not differ from the pre-adjusted OR value, which showed that the bias did not significantly affect the result. The Hedges' g for the ADG outcome, adjusted by using the trim-and-fill method, was likely to be inclined compared to the pre-adjusted Hedges' g of the outcome. Although a possible publication bias in ADG data was detected, vaccines are evaluated to help increase productivity against ED because an increase in ADG was still observed in the vaccinated pigs compared to that in the control from the adjusted data.

According to the results of network meta-analysis, higher posterior effect estimates and SUCRA scores were observed in mortality and ADG outcomes in pigs immunized with the plasmid-based DNA vaccine compared with those in the pigs immunized with other types of vaccines (Figs. 5b,c, 7b,c). With regard to clinical symptoms as the outcome, the pigs immunized with the plasmid-based DNA vaccine showed high posterior effect estimates and SUCRA scores, along with pigs vaccinated with only anti-serum or toxoid vaccines. (Fig. 6b,c). In the Bayesian network model, the plasmid-based DNA vaccine was considered the most effective, but 95% CrIs were formed relatively wide in forest plots of posterior effects for all outcomes, which may be caused by insufficient data (Figs. 5b, 6b, and 7b). However, plasmid-based DNA vaccines can obtain an effective antibody response against target antigens delivered by vectors and effectively defend against the virulence of extracellular bacteria (i.e., *E. coli*)^30,31^. In addition, given that the plasmid-based DNA vaccine showed increased effectiveness in all outcomes compared with that from any type of vaccine, the development of a plasmid-based DNA vaccine to prevent ED needs to be a high priority for future research.

The anti-serum vaccine directly acts as an antibody to block the movement of extracellular bacteria and their pathogenicity, and the recombinant toxoid or toxoid vaccines produce an antibody response against the toxin Stx2e, which mainly induces a humoral immune response^32^. In both mortality and clinical symptoms as the outcomes, the anti-serum vaccine showed increased posterior effect estimates and SUCRA scores compared with those in the controls (Figs. 5 and 6). Toxoid and recombinant toxoid vaccines also presented higher posterior effect estimates and SUCRA scores compared with those in non-vaccinated animals in the all outcomes (Figs. 5, 6, and 7). In particular, although the effectiveness of recombinant toxoid vaccine was not outstanding compared to that of other types of vaccines (Figs. 5c, 6c, and 7c), the posterior estimation for recombinant toxoid vaccine showed narrower and more precise 95% CrIs than those for other vaccine types (Figs. 5b, 6b, and 7b), providing valid evidence to support the immunogenicity. Currently, the recombinant toxoid vaccines can be easily accessed as they are commercially available, but caution is needed as there may be side effects from the adjuvants, which are necessary for inoculation with toxoid vaccines^33^. Overall, all types of vaccines were shown to be effective in preventing ED by inducing a defense, although the plasmid-based DNA vaccine was evaluated to have the highest effectiveness in reducing clinical symptoms and mortality and increasing ADG based on the current Bayesian network model.

Hedges’ g employed standardized mean difference using the value of standard deviation, allowing comparison between studies using different units of measurement^11^, which was used in the conventional meta-analysis in this study. However, Hedges’ g cannot be adapted for the Bayesian network model to evaluate posterior probabilities based on the prior probability and likelihood because of the limitations of the "gemtc" package in R program^34^. Alternatively, the ADG data used in this analysis were computed by employing the MD calculated in the unified measurement unit, kg/day. Further, the results of the network meta-analysis were visualized using star-shaped network models for all outcomes (Figs. 5a, 6a, and 7a). As there was no research that directly compared the effectiveness of each type of vaccine, the inconsistency of the network model was not evaluated. Owing to the limited number of studies on animal vaccines compared to those on human vaccines, direct comparison studies among the different types of vaccines were rarely found in our search strategy. Limitations are also inevitably induced in other parts of the process, as meta-analysis is generally conducted by extracting data from previously published research^35^. In this study, all relevant studies identified in the electronic database were evaluated without bias or language restrictions, and the incomplete retrieval of studies was unlikely to be a source of bias in this systematic review. However, the presence of unpublished eligible studies cannot be ignored, and it is also possible that relevant studies were missed in the electronic database despite the maximum refinement of our search strings. Furthermore, unintentional errors may have occurred during the data extraction process using the software WebPlotDigitizer Version 4.4 when extracting graphically reported data. To minimize this systematic error, two researchers independently attempted to extract and compare the data during the initial stage.

This systematic review and meta-analysis demonstrated that pigs vaccinated against ED have a lower risk of mortality and clinical symptoms, and show increased weight gain compared with that in non-vaccinated pigs. Although vaccinated pigs were observed to reduce fecal shedding, this could not be fully confirmed due to insufficient number of relevant studies. Among the different vaccine types, the plasmid-based DNA vaccine demonstrated a better effect than other types of vaccines based on the Bayesian network meta-analysis. Attention should be paid to the interpretation of the results in consideration of limitations, including the small number of studies, potential mediators, and potential bias within studies. Future research should focus on comparisons between different types of vaccines using large sample sizes to offer applicable information for clinical practice. The evidence provided in this meta-analysis and review will be useful for clinical veterinarians and researchers. It is expected that this review will not only guide clinical decision-making but also provide valuable information for future research design improvements for researchers.

## Materials and methods

### Study protocol and eligibility criteria

A protocol for systematic review and meta-analysis was prepared in advance according to the PRISMA-P guidelines (Supplemental Tables S4, S5, S6)^36,37^. The following components were included in the PICOS to assess the relevance of the primary studies identified in the search (Table 1).

- Population (P): As the target of the vaccine varies from sows to piglets, we defined the population of interest as pigs at any stage of production.
- Intervention (I): The eligible interventions were vaccine candidates against STEC or commercial vaccines for STEC (VEPURED, Ecoporc SHIGA). Studies that evaluated feed additives or antibiotics were excluded from this review.
- Comparator (C): Negative control, sham-vaccination, placebo, or other alternative treatments (including another vaccine) were eligible types of comparators.
- Outcomes (O): The following outcomes were assessed: mortality, clinical symptoms of ED, fecal shedding of STEC, and ADG. Humoral immune responses from antibody titers are considered as proxy indicators, wherein consistency and accuracy of results are not guaranteed due to high variance among the studies, leading to misinterpretation of the pooled effect sizes. Thus, antibody responses after vaccination against STEC were not eligible for this review. Further, postmortem or microbial findings, which can be subjectively evaluated, were excluded from the eligible outcomes.
- Study design (S): Randomized controlled trials to evaluate vaccine efficacy and field studies to evaluate vaccine effectiveness were included in this review. Ecological and descriptive observational studies and reviews were not suitable for this review. The publication year was limited from 1955, when the first research to identify the cause of ED was documented^38^.

**Table 1:** Eligibility criteria and search strings for assessing the effectiveness of edema disease vaccines in pigs.

	Inclusion criteria	Exclusion criteria
Study type	Controlled studies with natural or experimental STEC exposure Field studies with natural or experimental Shiga toxin-producing *Escherichia coli* (STEC) exposure Analytical observational studies Published 1955 or later	Ecological studies Descriptive observational studies Reviews
Patients	Pigs at any stage of production	Other animals except pigs
Intervention	Commercial vaccines or experimental vaccine candidates against edema disease strain of STEC	Vaccines unrelated to edema disease strain of STEC Assessment of additives or antimicrobials
Comparator	Negative control, sham-vaccination, placebo groups	No negative control, sham-vaccination, placebo groups
Outcomes	Mortality Clinical symptoms General symptoms Edema Neurological symptoms Average daily gain Fecal shedding of STEC	Did not assess vaccine effectiveness in pigs Postmortem findings Microscopic findings Antibody responses
Search strings		
(swine OR pig OR pigs OR piglet OR piglets OR sow OR sows OR hog OR hogs OR gilt OR gilts OR farrow OR nursery OR weaner OR postweaning OR post-weaning OR post weaning OR finisher) AND (*Escherichia coli* OR *E. coli* OR STEC) AND (F18 OR F18ab OR Shiga OR Stx2e OR Stx2eA OR Stx2eB) AND (Edema OR Oedema) AND (immuni* OR vaccin* OR interve* OR treatment OR effectiveness OR effect OR protect OR mitigat* OR control OR vepured OR ecoporc)		

### Search strategy and study selection

Eligible primary studies were identified in electronic databases using the search strings comprised five keywords: swine (population), STEC (intervention), virulence factors of STEC (intervention), ED (intervention), and vaccine (intervention and outcomes) (Table 1). Worldwide published research was accessed using PubMed, the Center for Agricultural Biosciences, Scopus, and Web of Science databases. Korean published research was accessed using the Research Information Sharing Service, ScienceOn, and DBpia databases. Gray literature was accessed using Google Scholar and ProQuest Dissertations and Thesis database. If it was difficult to access the full text, it was requested from the foreign research information center at Jeonbuk National University (http://www.fric.kr/user/centerMainView.do?centerId=jbnu). The search strings for finding relevant studies were adapted for each database, accounting for differences in indexing or functionality. The search dates were from 1955 to July 1, 2021. Language restriction was not applied to the search. Search results were uploaded to EndNote X9 (Clarivate, Philadelphia, PA, USA), and duplicates were removed. Two independent researchers assessed the eligibility of the studies. The first screening involved titles and abstracts, and the final screening involved full text. Disagreements were resolved by consensus or arbitration by an independent reviewer.

### Data extraction

The following data were extracted from eligible studies: name of author, publication year, study design, production phase, total number of pigs, commercialization of vaccine, vaccine type, vaccination route, dose, antigen in vaccine, type of comparator, number of pigs in each group, and relevant outcomes. Further, information on feeding crude protein or antimicrobials was extracted and used as confounding factors. In studies where multiple intervention groups existed, groups with similar characteristics were combined into one, as recommended by Cochrane^39^. For example, non-vaccinated and placebo groups were combined into the same control group and vice versa. In studies containing multiple intervention groups with different characteristics and one control group, the total number of the control group was evenly divided and assigned to each vaccinated group.

For relevant outcomes, mortality and clinical symptoms were measured as dichotomous variables. The main clinical symptoms of ED include general symptoms (loss of appetite, depression, and wasting), edema (palpebral or throat edema), and neurological symptoms (convulsion, rear-leg ataxia, extensor rigidity, lateral recumbency, tremors, paralysis, dyspnea, opisthotonos, and sudden death)^5,40^. Thus, if at least one clinical sign was observed in the animal after the challenge trial (i.e., experimental studies) or vaccination (i.e., field studies), it was placed in the event group. All outcomes of ADG and fecal shedding were extracted as continuous measures, carrying means, and standard deviations. If the mean and standard deviation were reported as graphs rather than numbers, the necessary data were extracted using WebPlotDigitizer Version 4.4 (Pacifica, CA, USA). The units of ADG and fecal shedding were unified as kg/day and log CFU/g, respectively. Attempts were made to contact the authors, if necessary, for additional information related to their study. In case there was no response from the authors, it was resolved through consensus among researchers.

### Risk-of-bias assessment

Two researchers independently assessed the quality of the primary studies selected for the meta-analysis by using the Animal Research: Reporting In Vivo Experiments (ARRIVE) guideline 2.0^41^. The domains consist of randomization, sample size, allocation concealment, blinding of participants and personnel, blinding of outcome assessment, incomplete outcome data, statistical methods, selective reporting of results, and other bias. Each study was assessed to be at either low, high, or unclear risk of bias for each domain. The final decision was made through consensus between the authors, whereas disagreements were resolved through the arbitration of an independent reviewer.

### Conventional meta-analysis

The meta-analysis for each outcome was conducted with “meta,” “metafor,” and “dmetar” packages in the statistical program R (version 4.1.1) and accompanying R-Studio (version 1.4)^11,42–45^. Data analysis was performed using random effect models with 95% CIs in consideration of the between-study variance. As the random effects variants based on Mantel–Haenszel and inverse variance methods have been employed as a method for pooling data for dichotomous and continuous outcomes, respectively, these methods were adopted based on each outcome type^25,42^. Heterogeneity was assessed by using τ^2^ estimate, Cochran’s Q test, and *I*^2^ value^46^. Based on the robustness of τ^2^ estimators, the Paule-Mandel and Restricted Maximum Likelihood procedures were selected to calculate the heterogeneity variance τ^2^ estimates of dichotomous and continuous outcomes, respectively^47–49^. Knapp-Hartung adjustment was applied in consideration of the uncertainty of the estimate due to between-study heterogeneity^50,51^. Nevertheless, once the high heterogeneity between studies was observed, the robustness of the pooled effect size and *I*^2^ value was evaluated using the leave-one-out method, and the influential outliers were determined through comprehensive evaluation of the robustness. The overall effect sizes of dichotomous and continuous data were reported as ORs and standardized mean differences (Hedges’ g), respectively. Continuity correction was performed by adding 0.5 for double-zero events in studies that clinical symptoms or death were not observed despite challenge with STEC. The results of data analysis were summarized as forest plots with 95% PIs and drapery plots including 90% and 99% CIs. Clinicians could identify what effectiveness is to be expected in future vaccinated pigs through the 95% PIs, and the reliability of the overall effect size can be determined through the 90% and 99% CIs^52^.

Subgroup analysis was conducted to investigate the source of heterogeneity, with predefined variables that could influence the outcomes (i.e., commercial availability, study type, type of vaccine, route of vaccine administration, growing stage of pigs, and ingestion of antimicrobial or crude protein). As investigations into heterogeneity do not yield useful results unless there are a significant number of studies, subgroup analysis was performed when there were over ten studies in a meta-analysis^25^. Publication bias was quantitatively assessed using contour-enhanced funnel plots and Egger’s regression test when ten or more studies were reported in a meta-analysis^26,53^. A trim-and-fill method was adapted to generate a non-biased effect size by imputing the number of missed studies in the plots^53^.

### Network meta-analysis

Network meta-analysis can be used to integrate indirect comparisons as well as direct comparisons to compare the effectiveness of multiple interventions simultaneously^54^. In Bayesian models, the posterior probabilities are estimated by using the data to update prior probabilities, such that a precise posterior probability can be obtained as the data accumulates^10^. Consequently, Bayesian network meta-analysis incorporates the advantages of network meta-analysis with those of Bayesian models, allowing simultaneous comparison of the posterior probabilities of the effects of multiple interventions. Here, we aimed to help future vaccine selection by using the Bayesian network model to provide more accurate posterior probabilities of vaccine effectiveness.

Bayesian network meta-analysis was performed using the “gemtc” package in the statistical program R (version 4.1.1) for outcomes reported in five or more studies^34,44,45,55,56^. As a prior model, a uniform distribution with an average of 0 and a variance of 5 was used (*σ ∼ U(0, 5*)). The MCMC simulation for generating posterior samples was implemented by Just Another Gibbs Sampler (JAGS) version 4.3.0 and “rjags” package in the software R version 4.1.1^30,31^. Four chains were simulated with 100,000 iterations, of which 5,000 'burn-in' iterations were discarded, and the values of every fifth iteration were extracted. The convergence of Bayesian models was assessed using the Gelman-Rubin plot.

The generated network model was visualized as a network plot, and the inconsistency and robustness of the network meta-analysis model were evaluated using the node-splitting method^57^. As the inconsistency assessment is performed by comparing the indirect effects calculated based on the network model with the direct effects, which is the result of actual studies, inconsistency of the network model could not be assessed in the absence of direct comparison between vaccines. In addition, posterior OR estimates for dichotomous data and posterior MD estimates for continuous data were presented as forest plots. The ranking of vaccines according to each outcome was visualized through the SUCRA with scores between 0 and 100%^58^. A SUCRA score closer to 100% means that the vaccine is more likely to be top-ranked and should therefore be chosen as a priority.

## Supplementary Information

Supplementary Legends.

Supplementary Figure S1.

Supplementary Table S1.

Supplementary Table S2.

Supplementary Table S3.

Supplementary Table S4.

Supplementary Table S5.

Supplementary Table S6.

## Data Availability

All data generated or analysed during this study are included in this published article (and its Supplementary Information files).
