# Supplementary material for: Systematic review and network meta-analysis to compare vaccine effectiveness against porcine edema disease caused by Shiga toxin‐producing Escherichia coli
Source: Sci Rep. 2022 Apr 19;12:6460. doi: 10.1038/s41598-022-10439-x (PMC9019103; doi:10.1038/s41598-022-10439-x)
Supplement: Supplementary file 1 — Supplementary Legends. [file 41598_2022_10439_MOESM1_ESM.docx]

**Supplementary information**

**Supplemental Figure S1.** Influence analysis of the average daily gain (ADG) data. Results were computed by omitting each study (left) and informed on the right. The green interval represents the 95% confidence interval for the pooled effect size.

**Supplemental Table S1.** Characteristics of eligible studies for assessment of vaccine effectiveness against porcine edema disease.

**Supplemental Table S2.** Results of influence analyses for average daily gain (ADG) in vaccinated pigs.

**Supplemental Table S3.** Results of a subgroup analysis of average daily gain (ADG) of vaccinated pigs.

**Supplemental Table S4.** PRISMA-P checklist for assessment of vaccine effectiveness against porcine edema disease.

**Supplemental Table S5.** PRISMA-P abstract checklist for assessment of vaccine effectiveness against porcine edema disease.

**Supplemental Table S6.** PRISMA-P NMA checklist for assessment of vaccine effectiveness against porcine edema disease.
