# Supplementary material for: Systematic review and network meta-analysis to compare vaccine effectiveness against porcine edema disease caused by Shiga toxin‐producing Escherichia coli
Source: Sci Rep. 2022 Apr 19;12:6460. doi: 10.1038/s41598-022-10439-x (PMC9019103; doi:10.1038/s41598-022-10439-x)
Supplement: Supplementary file 2 — Supplementary Figure S1. [file 41598_2022_10439_MOESM2_ESM.pptx]

## Slide 1
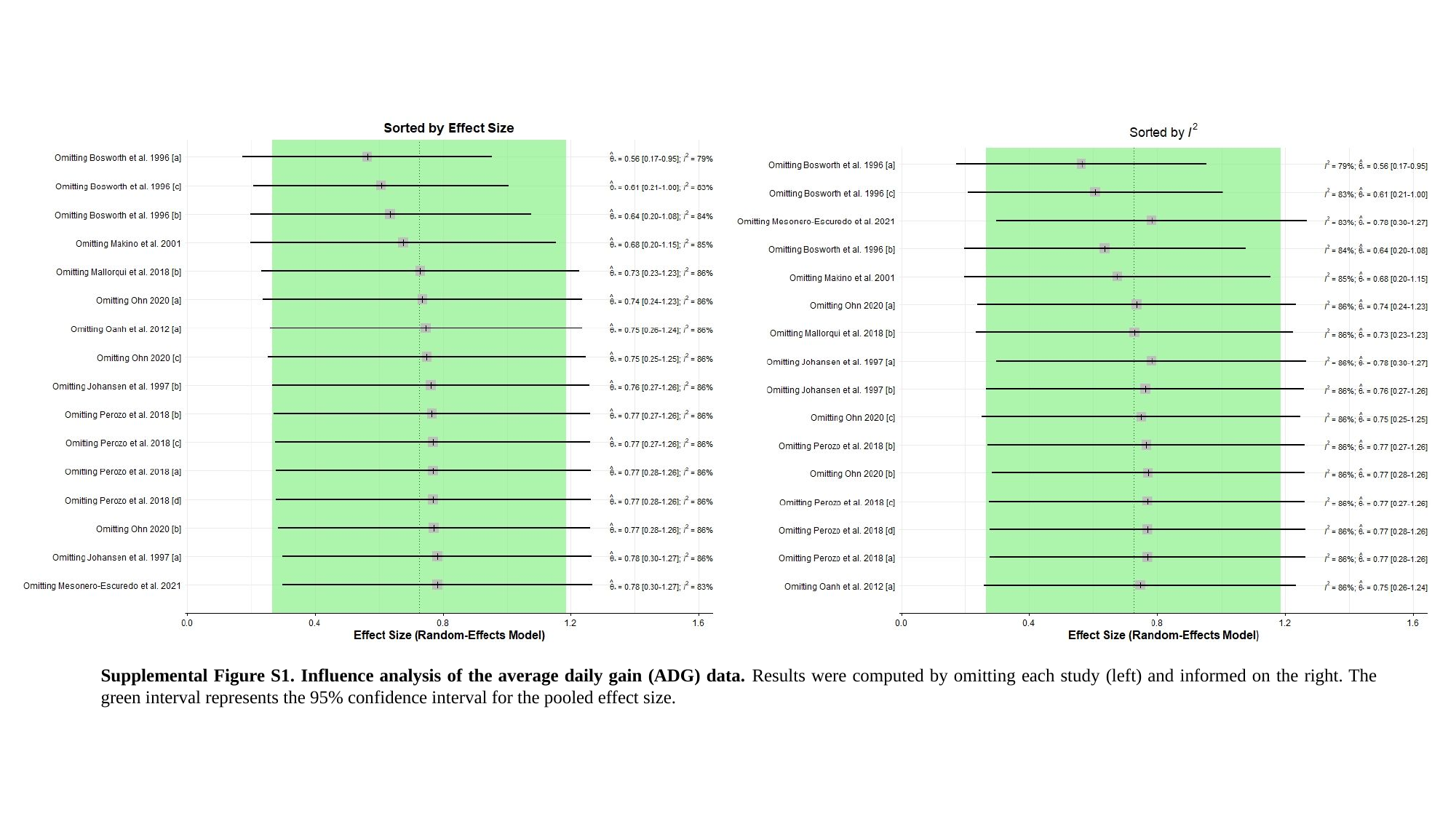

Supplemental Figure S1. Influence analysis of the average daily gain (ADG) data. Results were computed by omitting each study (left) and informed on the right. The green interval represents the 95% confidence interval for the pooled effect size.
